# An Eocene orthocone from Antarctica shows convergent evolution of internally shelled cephalopods

**DOI:** 10.1371/journal.pone.0172169

**Published:** 2017-03-01

**Authors:** Larisa A. Doguzhaeva, Stefan Bengtson, Marcelo A. Reguero, Thomas Mörs

**Affiliations:** 1 Department of Palaeobiology, Swedish Museum of Natural History, Stockholm, Sweden; 2 Division Paleontologia de Vertebrados, Museo de La Plata, Paseo del Bosque s/n, B1900FWA, La Plata, Argentina; University of California, UNITED STATES

## Abstract

**Background:**

The Subclass Coleoidea (Class Cephalopoda) accommodates the diverse present-day internally shelled cephalopod mollusks (*Spirula*, *Sepia* and octopuses, squids, *Vampyroteuthis*) and also extinct internally shelled cephalopods. Recent *Spirula* represents a unique coleoid retaining shell structures, a narrow marginal siphuncle and globular protoconch that signify the ancestry of the subclass Coleoidea from the Paleozoic subclass Bactritoidea. This hypothesis has been recently supported by newly recorded diverse bactritoid-like coleoids from the Carboniferous of the USA, but prior to this study no fossil cephalopod indicative of an endochochleate branch with an origin independent from subclass Bactritoidea has been reported.

**Methodology/Principal findings:**

Two orthoconic conchs were recovered from the Early Eocene of Seymour Island at the tip of the Antarctic Peninsula, Antarctica. They have loosely mineralized organic-rich chitin-compatible microlaminated shell walls and broadly expanded central siphuncles. The morphological, ultrustructural and chemical data were determined and characterized through comparisons with extant and extinct taxa using Scanning Electron Microscopy/Energy Dispersive Spectrometry (SEM/EDS).

**Conclusions/Significance:**

Our study presents the first evidence for an evolutionary lineage of internally shelled cephalopods with independent origin from Bactritoidea/Coleoidea, indicating convergent evolution with the subclass Coleoidea. A new subclass Paracoleoidea Doguzhaeva n. subcl. is established for accommodation of orthoconic cephalopods with the internal shell associated with a broadly expanded central siphuncle. Antarcticerida Doguzhaeva n. ord., *Antarcticeratidae* Doguzhaeva n. fam., *Antarcticeras nordenskjoeldi* Doguzhaeva n. gen., n. sp. are described within the subclass Paracoleoidea. The analysis of organic-rich shell preservation of *A*. *nordenskjoeldi* by use of SEM/EDS techniques revealed fossilization of hyposeptal cameral soft tissues. This suggests that a depositional environment favoring soft-tissue preservation was the factor enabling conservation of the weakly mineralized shell of *A*. *nordenskjoeldi*.

## Introduction

The present-day shelled coleoid cephalopods, comprising the two genera *Spirula* and *Sepia*, have a long evolutionary history with indisputable earliest records of rostrum-bearing coleoids in the Early Carboniferous [1, 2]. Recent recognition of the Early–Late Carboniferous bactritoid-like coleoids [3–9] supports the idea that Coleoidea originated from the Bactritoidea [10–12]. The Late Carboniferous *Shimanskya* from Western Texas, Brewster Country, Marathon Basin; USA, has a *Spirula*/*Sepia* shell-wall type characterized by absence of nacreous layer. This shell-wall type has been earlier known in the fossil spirulid genus *Adygeya* from the Early Cretaceous of north-western Caucasus [13] and is atypical for the externally shelled cephalopods, in which a nacreous layer forms the bulk of the shell-wall thickness [14–20]. Thus, *Shimanskya* demonstrates the evolutionary stability of the *Spirula*/*Sepia* shell-wall type through a period of about 330 million years. Another Late Carboniferous coleoid genus, *Donovaniconus* (order Donovaniconida), retained a shell wall with nacreous layer, as in bactritoids, but secreted a rostrum-like sheath upon it and an ink sac, which is not the case with the bactritoids [4]. The chemical data that would clarify whether the original shell composition in *Shimanskya* and *Donovaniconus* was organic-rich, as in *Sepia* and *Spirula*, are yet absent. However, data are available from the cuttlebone of the Eocene cuttlefish *Mississaepia* to show that shell wall and septa were rich in a chitin-compatible component and had a high content of nitrogen [21, 22]. The extant *Sepia* secretes an ultra-lightweight, high-stiffness organic-rich biomaterial that efficiently maintains neutral buoyancy at considerable habitation depths [23–27]. In various ectocochleate cephalopods, irrespective of their systematic affiliation, the wall typically consists of the outer prismatic, nacreous and inner prismatic layers [14–20] but rare forms among them have additional layers on the standard shell wall [28–30]. These layers imply a capability to stretch the mantle onto the external shell surface, producing an internally shelled condition that, at first glance, is similar to that in *Spirula* and *Sepia*.

The present paper reports a new subclass Paracoleoidea Doguzhaeva n. subcl. It is based on a unique Early Eocene straight-shelled cephalopod, *Antarcticeras nordenskjoeldi* n. gen., n. sp., from Seymour Island, Antarctic, that is characterized by thin microlaminated organic-rich shell wall, non-biomineralized septa, a central broadly expanded siphuncle, and an unusually broad septal foramen. The shell wall characteristics indicate the internalization of the shell. The central siphuncle illuminates the ancestry of a new subclass from the subclass Orthoceratoidea. The phylogenetic significance of the unique Eocene cephalopod from Antarctica is discussed.

## Materials and methods

### Material

Two specimens of fragmentary preserved straight shells from the La Meseta Formation [31–35] on Seymour Island, Antarctica, are examined. The age of the shells is dated as Ypresian, Early Eocene (for Geological setting and Palaeoenvironment, see S1 Text [36–53]). They are stored in the collections of the Department of Palaeobiology at the Swedish Museum of Natural History under depository numbers NRM-PZ Mo 167764, NRM-PZ Mo 167765 and are available upon request. These shells are described as *Antarcticeras nordenskjoeldi* n. gen., sp. (see Systematic Paleontology herein). A coiled nautilid, *Euciphoceras* sp. (NRM-PZ Mo 167766), from the La Meseta Formation on Seymour Island is examined with the purpose to evaluate the ultrastructural preservation of the cephalopod shell material in these rocks (see Supporting materials). Recent beach-collected specimens of *Spirula spirula* from Cuba (NRM-PZ Mo 167767) and *Sepia officinalis* from south Portugal (NRM-PZ Mo 1677648) are studied for comparison of shell wall composition between modern coleoids and *A*. *nordenskjoeldi*. The specimen NRM- PZ Mo 167764,which is cut longitudinally, provided detailed information on shell structures and ultrastructures of *A*. *nordenskjoeldi*. The specimen NRM-PZ Mo 167765 is studied in surface view and gave additional evidence of enrichment of the shell wall with organic material.

### Methods

The specimens were analyzed using light microscopes and SEM/EDS. Complete description of the material and methods are given in the Supporting Online Material (S1 Text).

### Nomenclatural Acts

The electronic edition of this article conforms to the requirements of the amended International Code of Zoological Nomenclature, and hence the new names contained herein are available under that Code from the electronic edition of this article. This published work and the nomenclatural acts it contains have been registered in ZooBank, the online registration system for the ICZN. The ZooBank LSIDs (Life Science Identifiers) can be resolved and the associated information viewed through any standard web browser by appending the LSID to the prefix “http://zoobank.org/”. The LSID for this publication is: urn:lsid:zoobank.org:pub:955FB82C-06BA-4CA8-8E63-C1145DC31376. The electronic edition of this work was published in a journal with an ISSN, and has been archived and is available from the following digital repositories: PubMed Central, LOCKSS, DiVA (http://www.diva-portal.org/).

## Results

### Scanning Electron Microscopy/Energy Dispersive Spectrometry (SEM/EDS)

**Fig 1:**
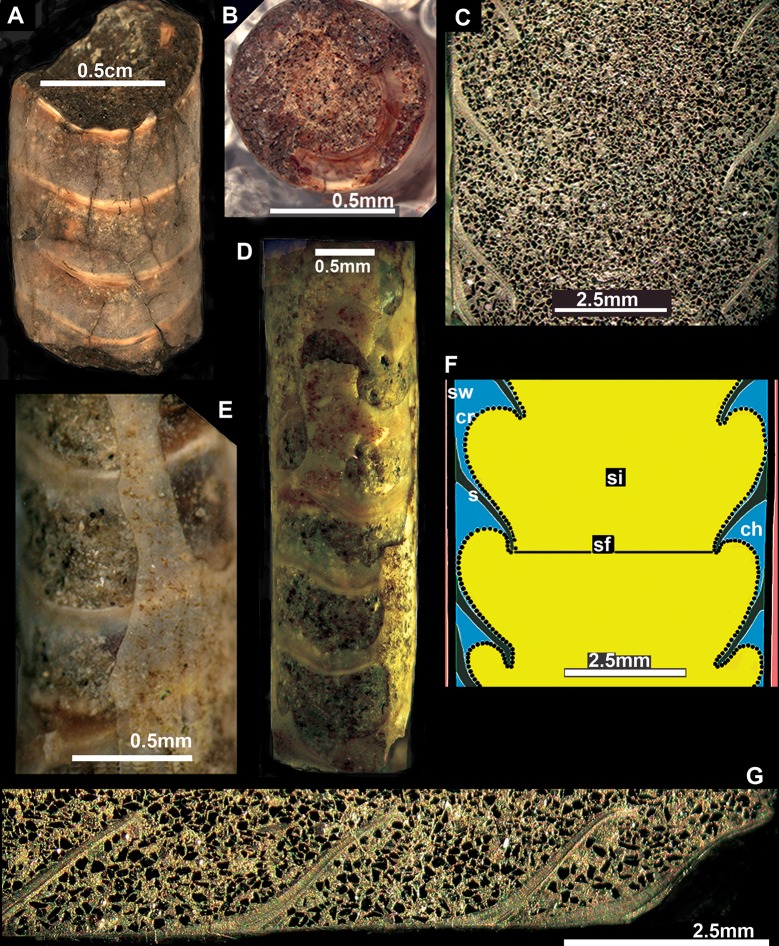
Shell morphology of *Antarcticeras nordenskjoeldi*. A–C, G, NRM-PZ Mo 167764; D, E, NRM-PZ Mo 167765; Early Eocene (Ypresian); Seymour Island, Antarctica. A, An orthoconic shell fragment with three complete chambers, thin semi-transparent shell wall and lobate sutures. B, Partially exposed septum and broad central septal neck at the adapical shell fracture. C, Deep septa and broad central septal necks indicative of a central broadly expanded siphuncle; longitudinal section. D, An orthoconic shell with lobate sutures. E, Close-up of D, thin semi-transparent shell-wall with inserted grains of a matrix indicative of a loosely mineralized shell wall. F, Sketch of internal shell structure. G, Close-up of left side of shell on C. Cr, connecting ring (dot line); ch, chamber outside siphuncle (blue); s, septum (green); sf, septal foramen; si, siphuncle (yellow); sw, shell wall (black).

**Fig 2:**
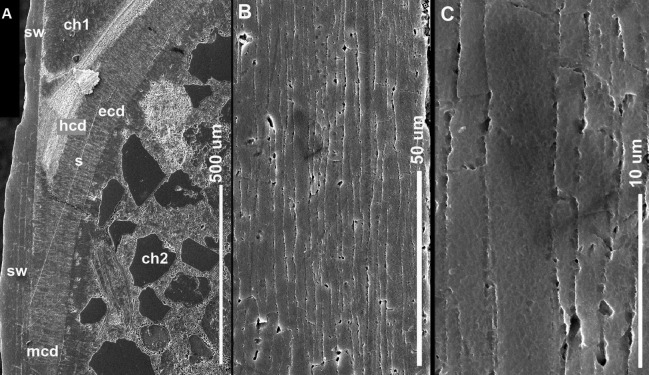
Shell ultrastructure *in Antarcticeras nordenskjoeldi*. NRM-PZ Mo 167764. Early Eocene (Ypresian); Seymour Island, Antarctica. A, Microlaminated shell wall (to the left) and prismatic cameral deposits on each side of septum; B, C, Close-up of A; ch1, ch2, the first and the second preserved cameras; ecd, episeptal cameral deposits; hcd, hyposeptal cameral deposits; mcd, mural cameral deposits; s, septum; sw, shell wall.

**Fig 3:**
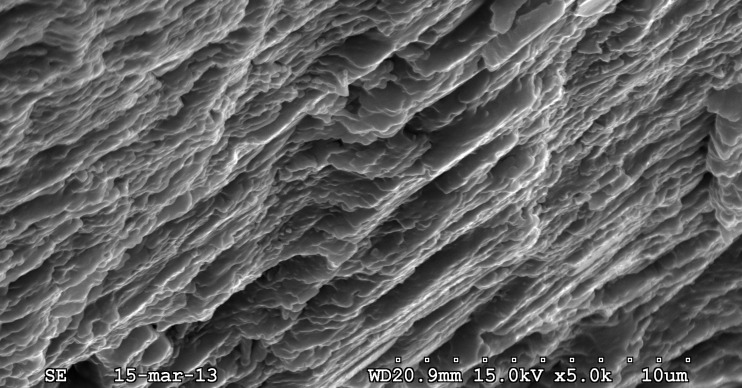
Antarcticeras nordenskjoeldi. NRM-PZ Mo 167764. Early Eocene (Ypresian); Seymour Island, Antarctica. Microlaminated shell wall; longitudinal shell section; outer shell surface to the left; inner shell surface to the right; direction towards shell aperture is top right corner.

**Fig 4:**
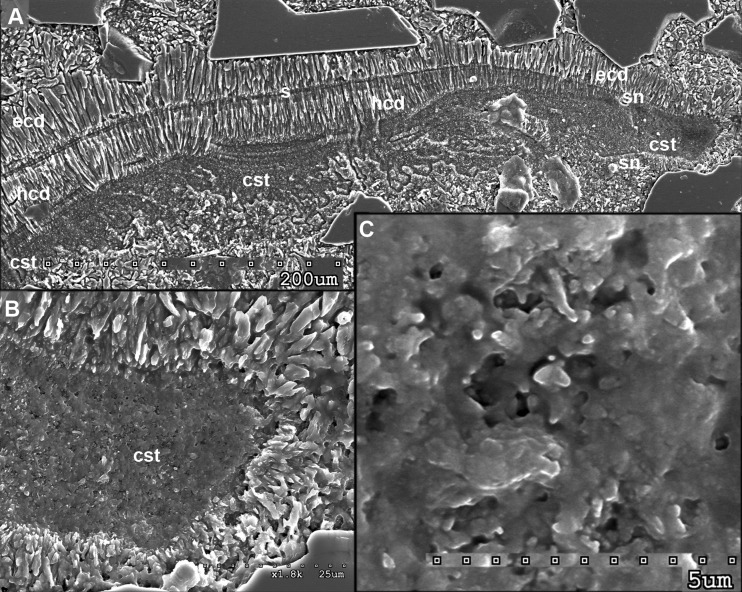
Antarcticeras nordenskjoeldi. NRM-PZ Mo 167764. Early Eocene (Ypresian); Seymour Island, Antarctica. A, Prismatic epi- and hyposeptal cameral deposits and microlaminated, microgranular cameral soft tissues; median shell section. B, C, Close-up of A. Cst, cameral soft tissue remains; ecd, episeptal cameral deposits; hcd, hyposeptal cameral deposits; s, septum; sn, suborthohoanitic septal neck.

**Fig 5:**
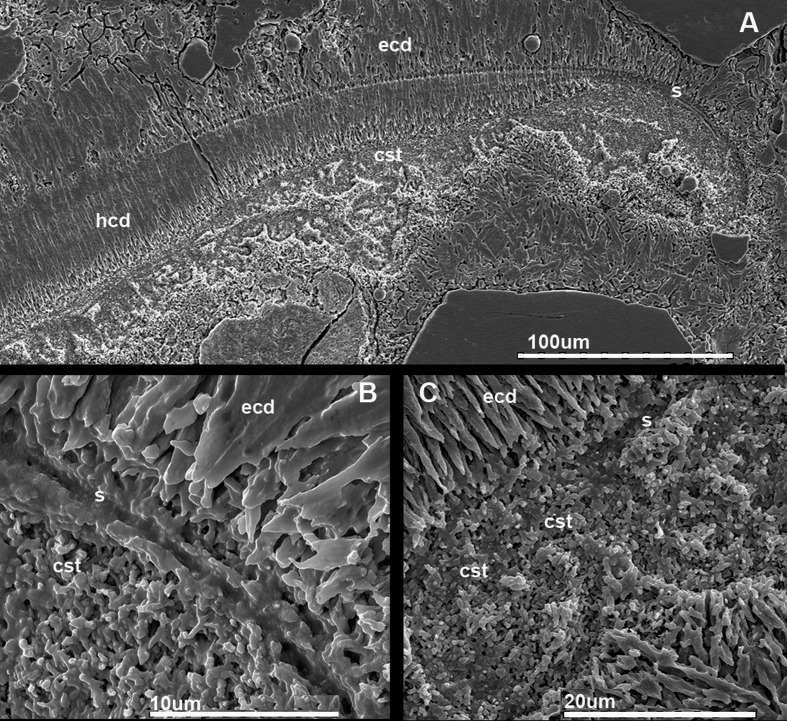
Antarcticeras nordenskjoeldi. NRM-PZ Mo 167764. Early Eocene (Ypresian); Seymour Island, Antarctica. Hyposeptal cameral soft tissues. A, general view. B, C, enlargements of A to show a microglobular ultrastructure of cameral soft tissues, median shell section. Cst, cameral soft tissue; ecd, episeptal cameral deposits; hcd, hyposeptal cameral deposits.

**Fig 6:**
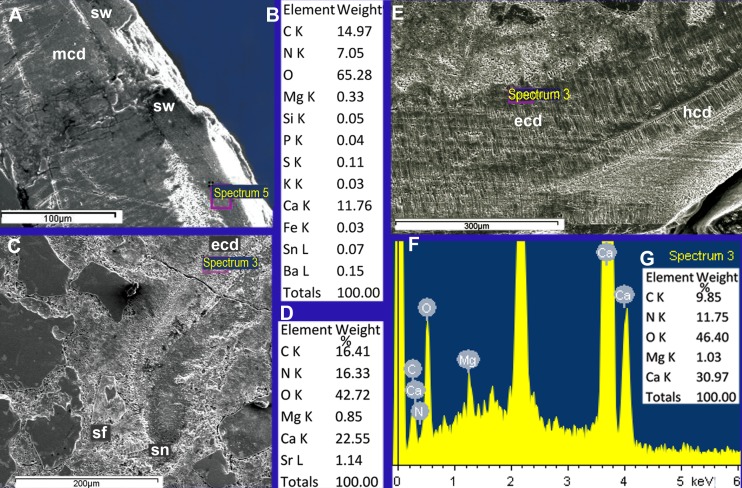
Antarcticeras nordenskjoeldi. (NRM-PZ Mo 167764). The organic-rich shell composition evidenced by high content of nitrogen in the shell wall (A, B) and episeptal cameral deposits at places near septal neck (C, D) and shell wall (E–G).

Analyses of *A*. *nordenskjoeldi* under SEM/EDS revealed a unique combination of morphological, ultrastructural and chemical traits; they are: (1, 2) chambered, longiconic conch; (3, 4, 5) thin microlaminated shell wall, having no nacreous layer; (6, 7) a central/or slightly sub-central, broadly expanded siphuncle; (8) notably large (0.6 shell diameter) septal foramen, 2–6 times wider than that of all other known cephalopods; (9, 10, 11) deep, thin, organic septa; (12, 13) thin suborthochoanitic septal necks; (14, 15) thin non-mineralized connecting rings; (16) inorganic-organic mural, epi- and hyposeptal cameral deposits; (17) hyposeptal cameral soft tissues; (18) lack of endosiphuncular deposits; (19, 20) lack of a rostrum and pro-ostracum; (21) irregular mineralization of the shell wall evidenced by the variable content of calcium (5.4% -27.8%); (22) inorganic-organic shell wall composition indicated by high content of nitrogen (up to 7. 8%) and additionally by lower (less than 1%) content of manganese, iron, nickel, copper, zinc, barium, thallium and lead (Figs 1A–1G, 2A–2C, 3, 4A–4C, 5A–5C and 6A–6G; S1, S2A, S2B, S3, S4A, S4B, S6A, S6B and S8A–S8H Figs; Table 1).

**Table 1:** *Antarcticeras nordenskjoeldi*; Early Eocene; Seymour Island, Antarctica.

Element	Matrix in shell	Shell wall	Mural cameral deposits	Episeptal cameral deposits	Hypo-septal cameral deposits	Soft tissue remains, organic debris	Mandible-like plate in adoral camera
Carbon	9.0–33.2	15.0–54.4	9.8–15.7	9.8–47.0	16.7–45.3	11.2–33.8	10.4–15.8
Nitrogen	0.0–8.0	0.0–7.0	0.0–13.5	0.0–16.3	no data	0.0–6.1	10.8
Oxygen	45.7–62.7	37.5–69.2	52.4–65.6	32.7–59.5	39.9–55.7	42.2–67.0	47.0–52.7
Sodium	0.0–5.9	0.0–1.0	0.0–0.2	0.0–0.3	0.0–0.1	0.0–3.7	no data
Magnesium	0.0–7.5	0.0–1.0	0.0–1.4	0.4–1.0	0.0–1.0	0.0–4.1	0.9–1.2
Aluminium	0.0–8.8	0.0–4.0	no data	no data	no data	0.0–5.2	no data
Silicon	0.1–30.3	0.0–14.7	0.0–0.1	no data	no data	0.0–11.2	no data
Phosphorus	0.0–0.4	0.0–1.6	0.0–0.7	no data	0.0–0.1	0.0–0.2	no data
Sulfur	0.0–0.3	0.0–0.2	no data	0.0–0.2	no data	no data	no data
Potassium	0.0–1.6	0.0–0.1	0.0–0.1	0.0–1.1	0.0–0.4	0.0–3.0	no data
Calcium	0.1–27.8	1.92–28	10.3–31.0	15.5–39.7	14.2–26.7	0.0–39.7	30.8–33.6
Manganese	0.0–0.1	0.0–0.2	0.0–0.1	no data	no data	no data	no data
Iron	0.0–6.3	0.0–0.2	0.0–0.1	0.0–0.4	0.0–0.2	0.0–7.6	no data
Nickel	0.0–0.1	0.0–0.1	0.0–0.5	no data	0.0–0.5	no data	no data
Copper	0.0–1.0	0.0–0.9	no data	0.0–1.3	0.0–0.4	no data	no data
Zinc	0.0–0.7	0.0–0.8	0.0–0.7	0.0–0.4	0.0–0.8	0.0–0.0	no data
Bromine	0.0–1.6	0.0–2.1	no data	no data	no data	no data	no data
Strontium	0.0–1.3	0.0–0.7	0.0–0.5	0.0–1.1	0.0–0.5	0.0–0.2	no data
Tin	0.0–0.4	0.0–07	no data	no data	no data	0.0–0.7	no data
Barium	0.06	0.0–0.3	0.0–0.3	0.0–1.1	0.0–0.3	0.0–0.6	no data
Thallium	0.0–0.4	no data	no data	no data	no data	0.0–0.7	no data
Lead	0.0–0.4	0.0–0.5	0.0–0.2	no data	0.0–0.2	0.0–0.4	no data

NRM-PZ Mo 167764, NRM-PZ Mo 167765. Energy-dispersive spectrometry data on elemental composition (in percent of total weight).

The prismatic mural, epi- and hyposeptal cameral deposits have, in comparison with the shell wall, higher average values of calcium (24% and 16.6%, respectively), carbon (27.6% and 18. 4%), nitrogen (7.05% and 4. 7%), magnesium (0.9% and 0.4%), potassium (0.9% and 0.2%), approximately similar values of oxygen (55.2% and 59.9%) and strontium (0.2% and 0.2%), but lower average values of copper (0.1% and 0.5%), zinc (0.5% and 0.7%), and lead (0.2% and 0.4%) (Fig 6A–6H; Table 1). The differences between the cameral deposits and the shell wall indicate an originally different chemical composition of these two shell structures. The contents of calcium and carbon, which are 1.5 times higher in epi- and hyposeptal cameral deposits than in the shell wall (Fig 6A–6H; Table 1), suggest stronger mineralization of the cameral deposits and largely organic composition of the shell wall. Besides, the epi- and hyposeptal cameral deposits have lower values of the elements known not to be used in the shells of extant cephalopods: copper, zinc, and lead (Table 1). This may indicate that the epi- and hyposeptal cameral deposits are less contaminated by metals than the shell wall. In recent coleoids soft tissues are easily contaminated by metals from the seawater [54–56], thus the occurrence of metals in the shell wall of *A*. *nordenskjoeldi* may be due to contamination of the organic matter. The tested orthocone is characterized by a high content of nitrogen (Fig 6A–6G; S8A–S8G Fig; Table 1): up to 8% in the shell wall and in the matrix in the siphuncle. However, the highest values of nitrogen are detected in mural, epi- and hyposeptal cameral deposits (12.8%) and the wing-like structure (10.8%). In contrast to the orthocone, nitrogen is missing in the shell wall of *Euciphoceras* (S5, S7A and S7B Figs).

Thus, the organic-rich composition of the microlaminated shell wall of *A*. *nordenskjoeldi* is evidenced by the variable content of calcium and high content of nitrogen (up to 8%), which is a reliable indicator of non-fossilized organic material [57]. Copper, zinc and lead in the shell-wall similarly suggest that it originally contained an amount of organic material.

### Comparison of shell structures between *A*. *nordenskjoeldi* and other recent and fossil cephalopods

#### Inorganic-organic (no nacre), or calcium carbonate (with nacreous layer) shell wall

**Fig 7:**
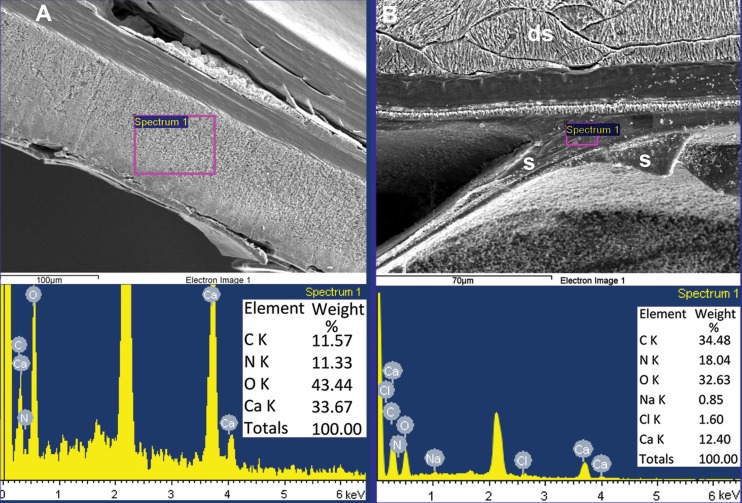
High content of organics indicated by high content of nitrogen (in percent of total weight) in (A) shell wall of *Spirula spirula*, Cuba, and (B) mural part of septum of *Sepia officinalis*, East Atlantic, Portugal.

**Fig 8:**
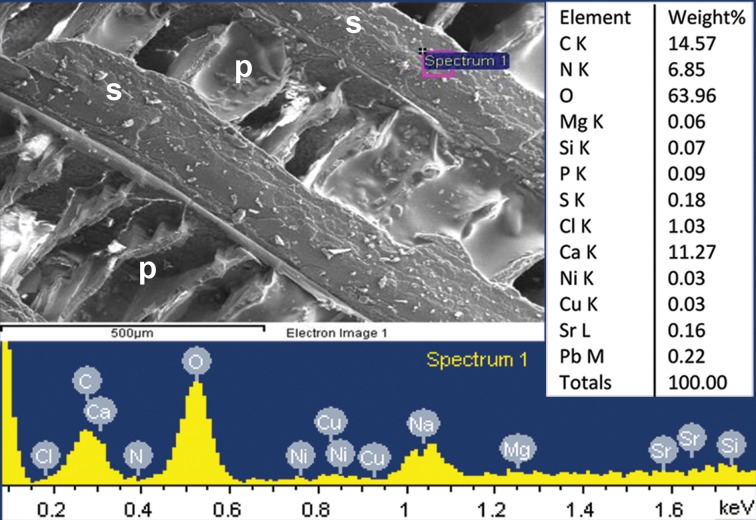
*Sepia officinalis*, East Atlantic, Portugal. High content of organics indicated by high content of nitrogen (in percent of total weight) in septum (EDS data); p, pillar zone; s, septum.

**Fig 9:**
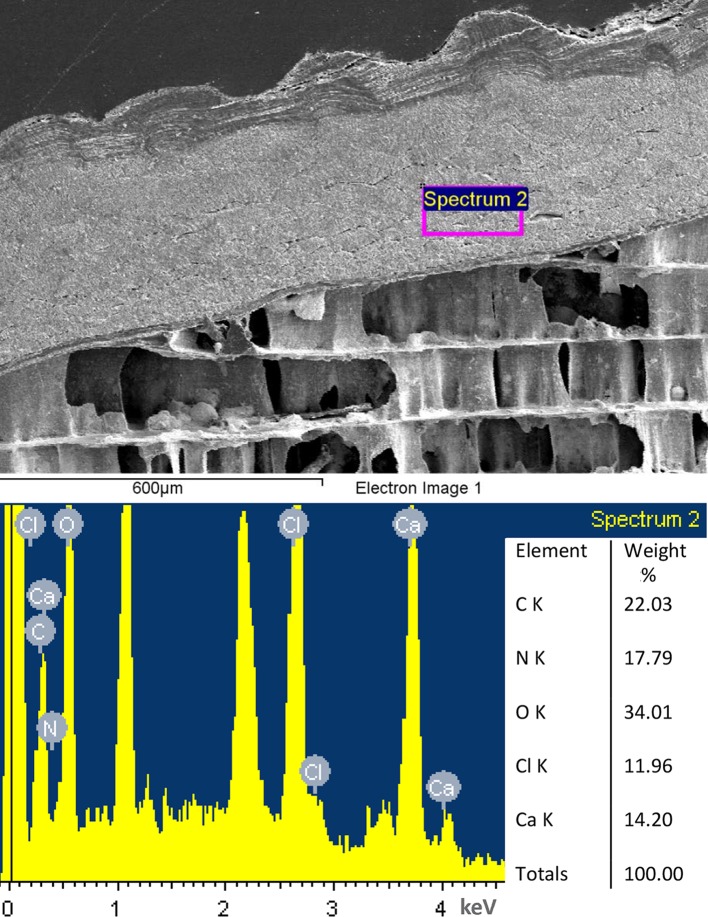
*Sepia officinalis*, East Atlantic, Portugal. High content of organics in dorsal shield indicated by high content of nitrogen (in percent of total weight) (EDS data).

Of the characters revealed in *A*. *nordenskjoeldi* (see above), the traits known in *Spirula* (Fig 7A; [13, 27]), *Sepia* (Figs 7B, 8 and 9); [13, 21–23]) and extinct coleoids [6, 9, 13, 21–25, 55–60] are: inorganic-organic shell wall material (Table 1); shell wall lacking nacreous layer (Figs 2A–2C and 3; S8A and S8B Fig), septa without columnar nacre (Figs 4A–4C, 5A and 5B; S3, S6A and S6B Figs) and with relatively high content of nitrogen, which is a reliable indicator of non-fossilized organic substance [21, 23, 57]. In contrast, the nautilid *Euciphoceras* (S5, S10 and S11 Figs) lacks the listed characteristics above.

Thus, with respect to its inorganic–organic shell-wall composition, *A*. *nordenskjoeldi* is comparable with the shelled coleoids *Spirula* (Fig 7A), *Sepia* (Figs 7B, 8 and 9; [22, 23]) and the Eocene *Mississaepia* [21, 22] but differs from the ectocochleates herein exemplified by the Eocene nautilid *Euciphoceras* sp. (S5, S10 and S11 Figs).

#### Organic-rich septa (no columnar nacre), or septa with columnar nacre

In having organic-rich septa (Figs 4A and 5A–5C; S3, S6A and S6B Figs), *A*. *nordenskjoeldi* is similar to the Eocene coleoid *Mississaepia* [21, 22], *Spirula* [61], *Sepia* [27] and the Late Carboniferous belemnoid *Donovanicous* [4], which have septa formed by lamellar-fibrillar nacre [27]. In contrast, the ectocochleate cephalopods have columnar nacreous septa [14–20].

#### Large or small septal foramen

A notably large (0.6 shell diameter) septal foramen (Fig 1C and 1F), 2–6 times bigger than that of all other cephalopods (S5 and S9 Figs), distinguishes the studied cephalopod from known coleoids as well as ectocochleates. In *Spirula* and in fossil coleoids, the septal foramen is about 0.1–0.2 shell diameter [61–66]; in Paleozoic ectocochleate cephalopods, including those with a broadly expanded central siphuncle (S9 Fig), it is 0.1–0.4 of the shell diameter [67, 68].

#### Central or marginal siphuncle

The central, or slightly sub–central, siphuncle (Fig 1A, 1C, 1F; S2A Fig) distinguishes *A*. *nordenskjoeldi* from *Sepia*, which has a siphonal zone instead of a tubular siphuncle, from the Late Cretaceous–Cenozoic belosaepiids, spirulirostrids, belemnoseids, missisaepiids and spirulids, in which a tubular siphuncle is marginal, or sub-marginal [13, 22, 62–66, 69–73]; from Mesozoic orthoconic cephalopods, with the exception of the Early Cretaceous orthocerid *Zhuravlevia insperata*, which has, however, a small (about 0.1–0.2 shell diameter) septal foramen and columnar nacre in shell wall and septa [18], and from Carboniferous coleoids (2–4, 6, 9).

Thus, in having a central/or slightly sub-central siphuncle, *A*. *nordenskjoeldi* is similar to the Ordovician–Early Cretaceous subclasses Orthoceratoidea and Actinoceratoidea but differs from the? Ordovician, Silurian–Late Permian Bactritoidea [49] and? Late Devonian, Early Carboniferous–Recent Coleoidea.

#### Broadly expanded or narrow siphuncle

The broadly expended siphuncle distinguishes *A*. *nordenskjoeldi* from *Spirula* and Late Carboniferous and Cretaceous spirulids, which have a narrow siphuncle [3, 13, 61, 63, 65].

The broadly expended siphuncle is a joint feature of *A*. *nordenskjoeldi* (Fig 1C and 1F), Orthoceratoidea and Actinoceratoiea (67, 68, 74). However, the endosiphuncular deposits that are weakly developed in some orthocerids but well developed in actinocerids (67, 68, 74) are missing in *A*. *nordenskjoeldi*. Therefore, there is more similarity of central siphuncle structure between *A*. *nordenskjoeldi* and orthocerids than between this genus and actinocerids.

## Discussion

The inorganic–organic shell-wall composition characterizes Recent *Spirula*, *Sepia*, and Eocene *Mississaepia* (Coleoidea), and is herein shown in *A*. *nordenskjoeldi;* hence these cephalopods are similar with respect to shell-wall material. In addition, a microlaminated shell ultrastructure apparently resulting from a high content of the chitin component in septa of *Spirula* and shell-wall and septa in *Sepia*, and of the chitin-comparable component in shell wall/septa in *Mississaepia*, is here shown to be a character of *A*. *nordenskjoeldi* as well. However, in having no external shell structure that would protect the thin laminated shell wall from the outside (Fig 1A, 1B, 1D, 1E; S2B, S4A and S4B Figs), *A*. *nordenskjoeldi* differs from three above-mentioned genera, as in *Sepia* (Fig 8B) and *Mississaepia* a dorsal shield coats the outer shell surface; in *Spirula*, this is the outer plate [13]. In the diverse Early Carboniferous–Late Cretaceous superorder Belemnoidea, a rostrum was secreted above the shell wall. Therefore, *A*. *nordenskjoeldi* has a microlaminated shell wall structure and *in vivo* organic-rich shell-wall composition, which indicate the internal shell position, as in coleoids, but in having no outer protective shell structure it is unique among cephalopods. It is worth noting that microlaminated organic-rich material is unknown in ectocochleates but typical for pro-ostraca of belemnoids and is therefore an attribute of the internal shell [56, 58–60]. The thin film-like non-mineralized septa of *A*. *nordenskjoeldi* (Figs 4A and 5A; S3 Fig) are most similar to those of the Eocene *Mississaepia* [22] and Recent *Sepia* (Figs 7B and 8). The prismatic cameral deposits enriched in organics are like those of belemnites [66]. The thin septal necks of *A*. *nordenskjoeldi* (Figs 4A, 5A and 6A; S6B Fig) are yet unknown in any other cephalopods. In *Spirula*, well-mineralized long holochoanitic septal necks consisting of lamellar-fibrillar nacre are strongly attached to the shell wall [61]. In S*epia*, the thin septa are achoanitic and formed by lamellar-fibrillar nacre [27]. In the Eocene sepiid *Mississaepia*, lamellar-fibrillar nacre lines thin chitinous septa [22].

Comparison of shell structures between *A*. *nordenskjoeldi* and other Recent and fossil cephalopods (see above) shows that the *Spirula/Sepia* type of shell characters, such as: organic-rich shell wall and septa, lack of nacreous layer in shell wall and lack of columnar nacre in septa, would have identified the Eocene *A*. *nordenskjoeldi* as a coleoid. However, the central, broadly expanded siphuncle in *A*. *nordenskjoeldi* speaks against membership of the subclass Coleoidea, thought to be derived from Palaeozoic bactritoids having narrow marginal siphuncles [10, 11]. Rather, these features suggest an origin in Paleozoic Orthoceratoidea possessing central or slightly sub-central siphuncles that are broadly expanded in some forms (S9 Fig) and free from endosiphuncular deposits.

The investigated Early Eocene orthoconic cephalopod *A*. *nordenskjoeldi* reveals the crisis characterized by "a prolonged interval during which the group virtually completely disappears from the paleontological record…the group became so restricted in taxonomic scope and in habitat that fossil representatives are great rarities or have not yet been identified at all" [74]. A new higher-order taxon is warranted for *A*. *nordenskjoeldi* as it shows non-complementary relationship between the morphological (central siphuncle) and ultrastructural (coleoid type shell wall and septum composition and ultrastructures) traits that suppresses its affiliation to any known high-level taxon in the class Cephalopoda. The placement of *A*. *nordenskjoeldi* either within a high-level taxon characterized with a marginal siphuncle and coleoid type shell wall composition (Coleoidea) or in a high-level taxon characterized by the *Nautilus*-like ectocochleate shell wall composition and ultrastructure would equally in every case conflict with a monophyly of the subclasses Coleoidea or Ortoceratoidea. The subclass Coleoidea Bather has been based on an assumed unique event of shell internalization in the evolution of cephalopods. The find of *A*. *nordenskjoeldi* indicates a second event of shell internalization indicative of a new taxon of equal rank to Coleoidea, herein described as subclass Paracoleoidea.

Based on *A*. *nordenskjoeldi*, after the Late Cretaceous mass extinction, there were three, rather than two, evolutionary lineages of internally shelled cephalopods: the *Spirula*, *Sepia* and *Antarcticeras* lineages. They are characterized by apparently different strategies of buoyancy regulation and, therefore, by different swimming activity. In the *Spirula* lineage, the buoyancy regulation strategy involves the usage of a narrow tubular siphuncle inherited from the bactritoid ancestors but enforced by means of elongation of the septal necks and strengthening of the septal neck/shell wall conjunction [61]. The living members of this lineage inhabited large depths diapasons–from several meters to about 1500 m. However, they have less broad geographic distribution and limited biodiversity than *Sepia*. In the *Sepia* lineage, the buoyancy regulation strategy apparently involved the transformation of a tubular siphuncle into a more effective “open” siphonal zone that allowed overcoming the limited capacities of the tubular siphuncle. This strategy is thought to be the most effective way of buoyancy regulation and is associated with extensive geographic distribution and high taxonomical diversity of living representatives [27]. In the *Antarcticeras* lineage, the buoyancy strategy evidently involved the usage of a broadly expanded siphuncle modified by means of significant broadening of the septal foramen.

The presence of a coleoid-like organic-rich shell wall in an Eocene straight-shelled cephalopod having central or slightly sub-central broadly expended siphuncle, seemingly derived from ectocochleate forms outside the bactritoids, prompts a reevaluation of the evolutionary history of Cephalopoda with respect to internalization of the shell, up till now thought to be a unique event in cephalopod evolution [10, 11, 63, 65, 70–75]. Internalization of the shell may have happened at least twice, followed by parallel or convergent evolution of shell ultrastructures and composition. The highly diverse Ammonoidea, extinct by the end of the Cretaceous, are generally accepted as a sister group to Coleoidea [10, 11], and although evidence is available for at least partial encroachment of the shell-secreting mantle onto the shell exterior in some genera [28–30], ammonoids are fundamentally ectocochleates, as are bactritoids and orthoceroids. The organic-rich shell, so far known as a coleoid-type shell structure resulting from the internalization of the shell, therefore turns out to be convergent. An alternative interpretation is that the condition of a narrow siphuncle and septal foramen, otherwise known to be stable in bactritoid and coleoid cephalopods, has been modified in the *Antarcticeras* lineage to produce a hitherto unknown type of internally shelled cephalopod that has overcome the limitations of the condition by expanding the siphuncle and septal foramen to allow for efficient buoyancy control. In this interpretation, the broadly expanded central siphuncle is a convergent feature with that of orthoceratoids. Of these two interpretations, the first one is, in our opinion, more probable. Convergence of habitat-related features is common among recent coleoids [76]. Thus, we suggest that a separate evolutionary lineage of internally shelled cephalopods, characterized by original inorganic–organic shell composition and broadly expanded central siphuncles, originated in the Late Paleozoic. It survived the end-Triassic and Cretaceous mass extinctions and lived on at least into the Early Eocene. Potential descendants of *A*. *nordenskjoeldi* among extant coleoids are not obvious; the lineage is most probably extinct. Although this lineage is so far indicated by a single Early Eocene *A*. *nordenskjoeldi* in Antarctica, the unique extra-ordinal combination of morphological, ultrastructural and chemical traits of the shell in this form motivates the erection of a new high-level taxon of equal rank with Coleoidea, which is described herein as subclass Paracoleoidea.

**Fig 10:**
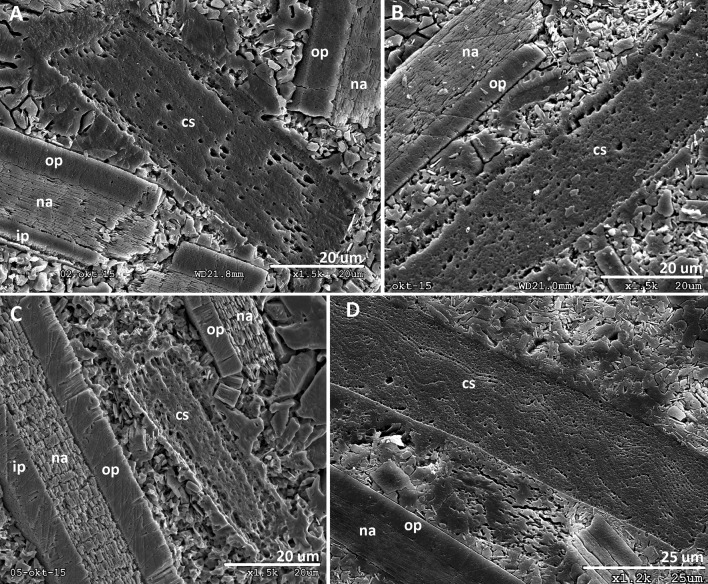
Structural differences between fossilized chitin of aptychi and aragonite shell wall of crushed juvenile ammonites preserved in the body chamber of the Aptian ammonite *Deshayesites*, Central Russia, Ulyanovsk. Ip, inner prismatic layer; na, nacreous layer; op, outer prismatic layer.

Recently described extinct forms [9, 22, 65, 69, 70, 72, 73] essentially increased fossil records of Coleoidea but yielded no taxa with a central siphuncle similar to *A*. *nordenskjoeldi*. *In vivo* inorganic-organic shell composition, in combination with physical and geochemical factors within the depositional environment that are favorable for soft-tissue fossilization, likely contributed to the preservation of *A*. *nordenskjoeldi*. The composition of the shell wall of *A*. *nordenskjoeldi* cannot yet be characterized in more detail; however, it is similar to the chitin in the aptychi, rather than to the shell wall, of the juvenile ammonites characterized by the aragonite shell composition (Fig 10).

## Systematic paleontology

Subclass Paracoleoidea Doguzhaeva n. subcl.

(Monotypic; diagnosis as for order.)

Order Antarcticerida Doguzhaeva n. ord.

(Monotypic; diagnosis as for family.)

Family Antarcticeridae Doguzhaeva n. fam.

urn:lsid:zoobank.org:act:30BC128F-3E2F-45CC-B384-E40F9FDFC464

(Monotypic; diagnosis as for genus.)

Genus *Antarcticeras* Doguzhaeva n. gen.

urn:lsid:zoobank.org:act:CC19DF36-765C-464E-8CB7-13667CE9F522

(Diagnosis as for type species.)

*Derivation of generic name*. After the Swedish South Polar Expedition vessel “Antarctic”.

Type and only species *Antarcticeras nordenskjoeldi* n. sp.

Species *Antarcticeras nordenskjoeldi* Doguzhaeva n. gen., n. sp.

urn:lsid:zoobank.org:act:1CDE8B47-2D2D-46B1-B745-BF9F3CD5FC66

(Figs 1A–1G, 2A–2C, 3, 4A–4C, 5A–5C and 6A–6C; S1–S3, S4A, S4B and S6A Figs; Table 1)

*Diagnosis*: Shell inorganic-organic, longiconic; angle of expansion 3–7°; smooth surface; rounded cross-section; no constrictions at sutures; shell wall thin, inorganic-organic, microlaminated, without nacreous layer; siphuncle central, or slightly sub-central, broadly expanded; septal foramen broad, rounded, about 0.6 of shell diameter; septal necks thin, non-mineralized, suborthochoanitic; connecting rings thin, non-mineralized; septa thin, supposedly chitinous, deeply concave; sutures transverse or indistinctly inclined; with two deep broad lobes; epi- and hyposeptal, and mural cameral deposits present.

*Derivation of specific name*: In honor of Otto Nordenskjöld, leader of the Swedish South Polar Expedition 1901–1903.

*Holotype*: NRM-PZ Mo 167764.

*Paratype*: NRM-PZ Mo 167765.

*Repository*. Swedish Museum of Natural History, Department of Palaeobiology; Stockholm.

*Locality and horizon*: NRM 8, below IAA 1/90 (Ungulate Site), Seymour Island, Graham Land, Antarctica. Cucullaea I shell bed, TELM 4, Ypresian, early Eocene, Paleogene.

## Conclusions

Our study presents the first evidence of an internally shelled fossil cephalopod with central siphuncle. SEM/EDS analyses of longiconic shells of the Eocene cephalopod *A*. *nordenskjoeldi* show an organic-rich microlaminated shell wall that indicates its secretion as an internal shell, like that of Recent *Sepia*. It is associated with thin non-mineralized septa. *A*. *nordenskjoeldi* indicates a previously unknown evolutionary lineage of internally shelled cephalopods with broadly expanded central or slightly sub-central siphuncle, herein described as subclass Paracoleoidea Doguzhaeva. This lineage supposedly originated within Late Paleozoic externally shelled Orthoceratoidea with central siphuncle broadly expanded between septa and free from endosiphuncular deposits. It evolved independently from the Bactritoidea/Coleoidea lineage. *A*. *nordenskjoeldi* shows that the endocochleate condition was not restricted to coleoids.

## Supporting information

S1 Text Materials and methods. Geological setting and depositional environment.
(PDF)

S1 Fig *Antarcticeras nordenskjoeldi* n. sp.; Early Eocene; Seymour Island, Antarctica. NRM–PZ Mo 167764. Lateral view through semi-transparent shell wall on four lobate sutures. Ch, chamber; ml, mandible–like structure; s, septum; sn, septal neck.
(TIF)

S2 Fig *Antarcticeras nordenskjoeldi* n. sp.; Early Eocene; Seymour Island, Antarctica. NRM–PZ Mo 167764. Transverse adapical (A) and adoral (B) shell fractures showing rounded shell cross section, broad septal neck attached to the shell wall on A and mandible–like structure on B.
(TIF)

S3 Fig *Antarcticeras nordenskjoeldi* n. sp.; Early Eocene; Seymour Island, Antarctica. NRM–PZ Mo 167764. The traces of thin organic structure-less septum squeezed between prismatic episeptal cameral deposits (right side) and granular hyposeptal cameral soft tissue remains (left side) at septal neck. Cst, cameral soft tissue remains; ecd, episeptal cameral deposits; s, septum; sn, septal neck.
(TIF)

S4 Fig *Antarcticeras nordenskjoeldi* n. sp.; Early Eocene; Antarctica, Seymour Island. NRM–PZ Mo 167765. A, Ventrolateral view on the partially exposed chambers of the phragmocone with extensive mural cameral deposits. B, Enlargement of Fig 1E to show remains of thin brown organic septa. C, chamber; mcd, mural cameral deposits; os, organic septum; sw. shell wall.
(TIF)

S5 Fig *Euciphoceras* sp.; Early Eocene; Antarctica, Seymour Island. NRM–PZ Mo 167766. Median shell section showing sub-central narrow siphuncle.
(TIF)

S6 Fig *Antarcticeras nordenskjoeldi* n. sp.; Early Eocene; Antarctica, Seymour Island. NRM–PZ Mo 167764–2. A, hyposeptal cameral soft tissues lining the adapical septal surface. B, enlargement of A showing thin suborthochoanitic septal neck and microglobular ultrastructure of hyposeptal cameral soft tissue. Ch, chamber; hcst, hyposeptal cameral soft tissue; s, septum; sn, cyrtochoanitic septal neck.
(TIF)

S7 Fig EDS data on elemental composition of a mandible-like structure on S2B. Note a peak of nitrogen indicative of non-fossilized organic material [57].
(TIF)

S8 Fig *Antarcticeras nordenskjoeldi* n. sp.; Early Eocene; Seymour Island, Antarctica. NRM–PZ Mo 167764. Energy-dispersive spectrometry data on chemical composition of shell wall (in percent of total weight).
(TIF)

S9 Fig A central, broadly expanded siphuncle of the Late Devonian pseudorthocerid *Arpaoceras*; R. Arpa, Armenia. Median shell section ([43]; modified). esd, episeptal cameral deposits; hsd, hyposeptal cameral deposits; s, septum; sf, siphuncular foramen; si, siphuncle; sn, septal neck.
(TIF)

S10 Fig *Euciphoceras* sp.; Early Eocene; Antarctica, Seymour Island. NRM–PZ Mo 167766. Columnar nacre of shell wall, median shell section.
(TIF)

S11 Fig *Euciphoceras* sp.; early Eocene; Antarctica, Seymour Island. NRM–PZ Mo 167766. Energy-dispersive spectrometer data on shell wall chemical composition. A, median shell section showing position of the spectrum taken. Ch, chamber of the phragmocone; s, septum; sw, shell wall. B, An energy-dispersive spectrometer graph showing spectrum 2 in A.
(TIF)
