# Supplementary material for: An Eocene orthocone from Antarctica shows convergent evolution of internally shelled cephalopods
Source: PLoS One. 2017 Mar 1;12(3):e0172169. doi: 10.1371/journal.pone.0172169 (PMC5332165; doi:10.1371/journal.pone.0172169)
Supplement: S1 Text — Geological setting and depositional environment. (PDF) [file pone.0172169.s001.pdf]

An Eocene orthocone from Antarctica shows convergent evolution of internally shelled cephalopods

Larisa A. Doguzhaeva[1*], Stefan Bengtson[1], Marcelo A. Reguero[2], Thomas Mörs[1]
[1]Department of Palaeobiology, Swedish Museum of Natural History, Stockholm, Sweden;
[2]Division Paleontologia de Vertebrados, Museo de La Plata, Paseo del Bosque s/n, B1900FWA, La Plata, Argentina

Supporting Materials

1. **Materials and Methods**

The paper is based on two Early Eocene longiconic shells (NRM–PZ Mo. 167764, NRM–PZ Mo. 167765) collected in Seymour Island, Antarctica, during the Argentine–Swedish Antarctic expeditions in 2011 and 2013. The additional studied material comprises a shell of the nautilid Euciphoceras sp. (NRM–PZ Mo. 167766), sampled from the lower Eocene in Seymour Island, Antarctica, during the Argentine-Swedish Antarctic expeditions 2011; a beach-collected shell of extant Spirula spirula (NRM–PZ Mo. 167767), Cuba, and a beach–-collected shell of extant *Sepia officinalis* (NRM–PZ Mo. 1677648), south Portugal.

The specimen NRM–PZ Mo. 167764 is a fragmentary shell, about 15 mm in length and 8 mm in diameter, with three complete and two partially destroyed cameras (Fig 2A–G; S1 and S2A, B Figs); each unbroken chamber is about 3 mm long. The adapical incomplete chamber shows partially exposed broad septal neck and septum (Fig-S2A). The incomplete adoral chamber contains a mandible-like, organic-rich structure formed by two long “wings”, with concentric growth lines, fused in the central nucleus, cylindrical in cross section (Fig 6A; S2B Fig). The total width of this structure is approximately equal to the shell diameter. The “wings” are similar to the wings of the chitinous lower mandible of some cephalopods; however, the central nucleus makes this interpretation uncertain. The structure contains non-fossilized organic material, as indicated by the presence of nitrogen (S6B Fig).

The specimen NRM-PZ Mo. 167765 is an 18 mm long shell fragment; shell diameters at the adapical and the adoral broken ends are 4 mm and 5 mm, respectively (Fig 1D; S4A and S4B Figs). This shell may represent an earlier ontogenetic stage than the first one. The two shells expose lobate sutures (Fig-1A, D, E; S4A–B Fig). Septa are indistinctly inclined dorsally. The external portions of the shell wall contain embedded grains of sand (Fig 1E; S4A, B Fig) that may evidence its weak original mineralization.

The specimen NRM–PZ Mo. 167766 is a medium size coiled shell with well-preserved outer surface and explored internal whorls (S5 Fig).

The studied specimens are housed at the Department of Palaeobiology, Swedish Museum of Natural History, Stockholm.

To understand the systematic position of herein described *A. nordenskjoeldi* n. sp., three categories of data have been obtained and analyzed: shell morphology, shell ultrastructure and chemical composition of shell material (Figs 1A–G; 2A–C; 4, 5A–C; 6A–C; 6A–G; S1; S2A, B; S3; S4; S5A, B; S7A–H Figs; Table 1). Initially, gross shell morphology was studied under light microscopes: Nikon, Wild Photomakroskop M 400, and Olympus SZX10. The specimen NRM–PZ Mo. 167764 was then cut in the median plane for study of the internal shell structures, particularly siphuncle morphology, shell wall and septa ultrastructures, and chemical composition. The two parts were examined by use of a scanning electron microscope, Hitachi S–4300, equipped with an energy dispersive spectrometer (EDS). The cut surfaces were polished, and one of them was etched with a 1%–3% solution of hydrochloric acid for 6–10 sec. and coated with gold for further ultrastructural and elemental analyses. EDS data were also taken from the external shell surface (NRM–PZ Mo. 167765). The chemical data were expected to clarify whether the shells have inorganic-organic composition, like the cuttlebone of *Sepia* or the

Spirula shell, or are mineralized, like the external shell of the nautilid *Euciphoceras* sp. from the same burial environment as the studied longicones. The *Euciphoceras* shell was examined under the SEM to give information on preservation of shell material in the burial environment relevant to the interpretation of the original shell material in the studied longicones (S5,-S10, S11 Figs). Elemental analyses were performed with an accelerating voltage of 15 kV, and energy calibration as measured on standard minerals of known accuracy were used. All elements were analyzed and no peaks were omitted. The specimen NRM–PZ Mo. 167765 (Fig 1D, E; S4A, B Fig) was examined using synchrotron based tomographic microscopy (SRXTM) at the TOMCAT beamline of the Swiss Light Source, Paul Scherrer Institute, Switzerland. The specimen (NRM–PZ 167764), studied in detail under the SEM, yielded data on the ultrastructural and chemical information. In these two specimens, distinguishing of the mural, epi- and hyposeptal cameral deposits with the aid of their prismatic ultrastructure and position on septal surfaces and shell wall (Figs-5A and 6A) helped recognize thin non-mineralized, most likely chitinous, septa (Figs 5A and 6A–C; S3; S5A, B Figs). The earlier observed thin non-mineralized chitinous septa of the Eocene coleoid *Mississaepia* [21, 22] were important for identifying the organic-rich septa in the studied orthocones. The preservation of cameral hyposeptal soft tissues (Figs 5A–C and 6A–C; S5A, B Fig) demonstrated that the burial environment could provide fossilization of organic-rich shell wall as well. The *Euciphoceras* shell (S5, S10 and S11 Figs) shows that the shell wall and septum ultrastructures are well preserved and show clear similarity to those of extant *Nautilus*, which suggested that the shell ultrastructures observed in the examined straight shells retain their original ultrastructural features, such as micro-banded shell wall. The morphological, ultrastructural and chemical data were compared with the available data on *Spirula* (Fig-8A, B; [13, 61]) and *Sepia* (Figs 8 and 9 [26, 27]). In addition, the data on the micro-laminated ultrastructure of the organic rich pro-ostracum in belemnoids [56, 58-60] as well as the micro-laminated ultrastructure of the chitinous gladius of extant squids (77) were used for the interpretation of the original material of the studied orthocones as well. The combination of morphological, ultrastructural and chemical approaches was useful in the assessment of the biology and systematic affiliation of *Antarcticeras*. In addition, the different groups of extant and extinct ecto- and endocochleate cephalopods, including extant *Spirula* (Fig 8A, B; [13, 35, 65]), *Sepia* (Figs 8 and 9; [21, 22–27, 54, 57]), the Eocene–Miocene *Vasseuria* [62] and *Spirulirostra* [64], Eocene sepiid *Missisaepia* [21, 22] and *Belosaepia* [69], Eocene coiled nautilid *Euciphoceras* (S5, S10 and S11 Figs), Early–Late Cretaceous coleoid *Naefia* [13, 40, 65], Cretaceous orthocerid *Zhuravlevia* [18], Late Carboniferous spirulid *Shimanskya* [3] and other Carboniferous bactritoid-like coleoids [4–9], Jurassic-Cretaceous belemnoids [47–49, 71] and Paleozoic orthoceroids (S9 Fig; [16-19, 67, 68, 74]) were included in the comparison.

2. **Geological setting and depositional environment**.

The marine sediments of the La Meseta Formation on Seymour Island are part of the James Ross Basin, a back-arc basin situated east of the Antarctic Peninsula [36–38The early to late Eocene/?earliest Oligocene La Meseta Formation [39, 44] rests as a northwest southeast trending incised valley fill on either Late Cretaceous or Paleocene units and represents deltaic, estuarine and shallow marine environments [41–44]. The La Meseta Formation is about 720 m thick and composed of mostly consolidated sandstones and mudstones with interbedded shell-rich conglomerates. Sadler [45] subdivided the formation into seven lithofacies units (Telm 1–7), and Marenssi et al. [41] introduced six erosionally based allomembers: Valle de Las Focas (= Telm 1), Acantilados (= Telm 2), Campamento (=Telm 3), Cucullaea I (= Telm 4–5), Cucullaea II (= Telm 5) and Submeseta (= Telm 6–7).

Specimen NRM–PZ Mo 167765 (collected in 2013) comes from a shell lens within the Acantilados Allomember (unit Telm 2 in [45]), deposited in a delta front setting [42]. The site is geographically and stratigraphically close to the site NRM 7 "Channel Site", which has

produced the oldest Antarctic mammal findings to date [46]. Specimen NRM PZ Mo 167764 (collected in 2011) derives from the Cucullaea I shell bed, which represents the basal part of the approximately 80 m thick Cucullaea I Allomember (unit Telm 4 in [45]). It is an up to 3 m thick shell bed dominated by the large, thick-shelled bivalve Cucullaea raea and darwinellid gastropods, but also very rich in vertebrate remains, especially shark teeth [47]. It is situated within the Antarctodarwinella nordenskjoldi Zone [44]. The Cucullaea I shell bed, which might actually consist "of a series of distinct but closely spaced shell bank beds and not a single continuous bank" [44: p. 42] can be traced at several sites along the southern, western and northern flanks of the northern Seymour Island Meseta. According to [44], the depositional environment of Telm 4 can be described as near shore, high-energy settings with locally protected environments. The latter can be assumed for our fossil site owing to the preservation of complete starfish. According to [42], Telm 4 represents an estuarine environment. Our Telm 4 fossil site is close to the stratigraphically higher Telm 5 locality IAA 1/90 (64°14'04.67"S; 56°39'56.38"W), from which the oldest animal sperms have been reported [48]. The age of the lower and middle part of the La Meseta Formation (Telm 1 to Telm 5) is controversial. Recently, Douglas et al. [49] proposed that the lower part of the La Meseta Formation (Telm 2 to Telm 4) is no older than the base of the middle Eocene (45 Ma, Lutetian), based on a magnetostratigraphically calibrated dinocyst biostratigraphic framework for the early Paleogene of the Southern Ocean [50]. This agrees with strontium isotope ratios (87Sr/86Sr) from bivalve shells that were used [34] to argue for a middle Eocene age (44.5 or 47.4 Ma). In contrast, strontium data [33] resolved a late early Eocene age (49–51 Ma). According to the new geological map [51] the base of the Acantillados Allomember (= Telm 2-3) is at 56.8 Ma and the base of Cucullaea II (= Telm 5) at 49.0 Ma, which would indicate an Early Eocene (Ypresian) age for our specimens. This older setting is confirmed by [52, 53] who correlated the mammal assemblage from Cucullaea I with the Patagonian locality Paso del Sapo, resulting in a latest early–earliest middle Eocene age (~49.5 Ma).
