# Supplementary figures and images for: An Eocene orthocone from Antarctica shows convergent evolution of internally shelled cephalopods
Source: PLoS One. 2017 Mar 1;12(3):e0172169. doi: 10.1371/journal.pone.0172169 (PMC5332165; doi:10.1371/journal.pone.0172169)

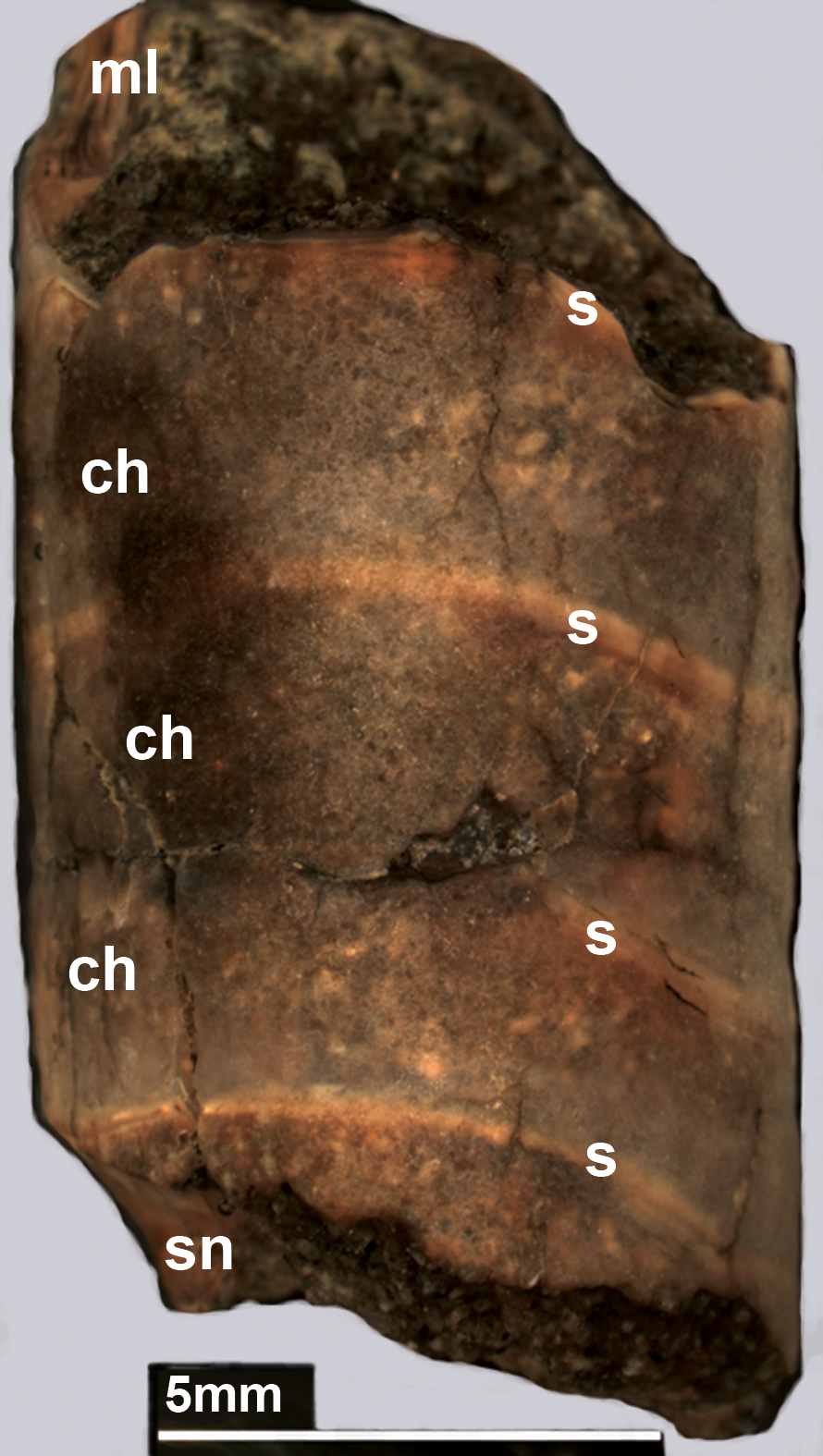

Supplement: S1 Fig — NRM–PZ Mo 167764. Lateral view through semi-transparent shell wall on four lobate sutures. Ch, chamber; ml, mandible–like structure; s, septum; sn, septal neck. (TIF) [file pone.0172169.s002.tif]

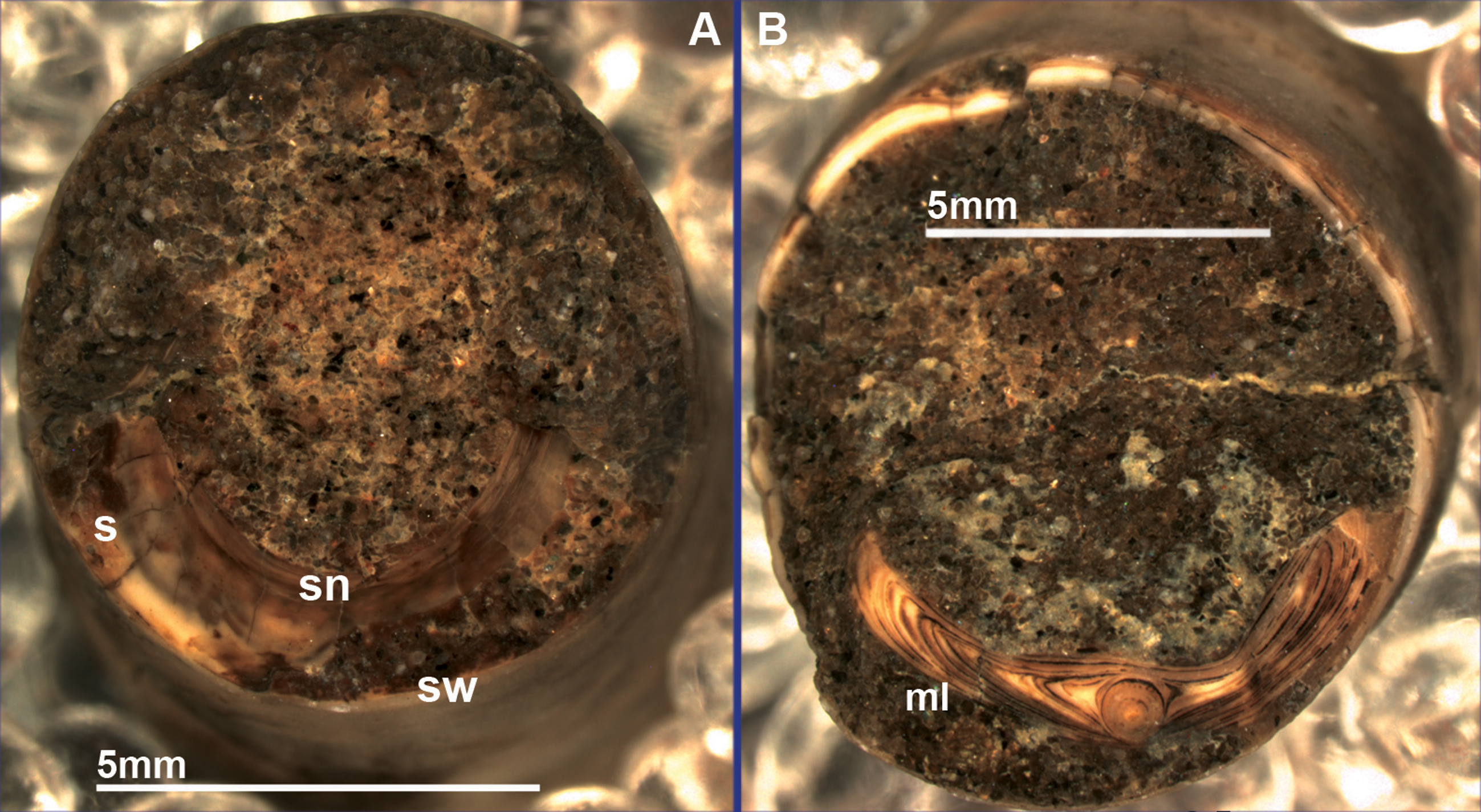

Supplement: S2 Fig — NRM–PZ Mo 167764. Transverse adapical (A) and adoral (B) shell fractures showing rounded shell cross section, broad septal neck attached to the shell wall on A and mandible–like structure on B. (TIF) [file pone.0172169.s003.tif]

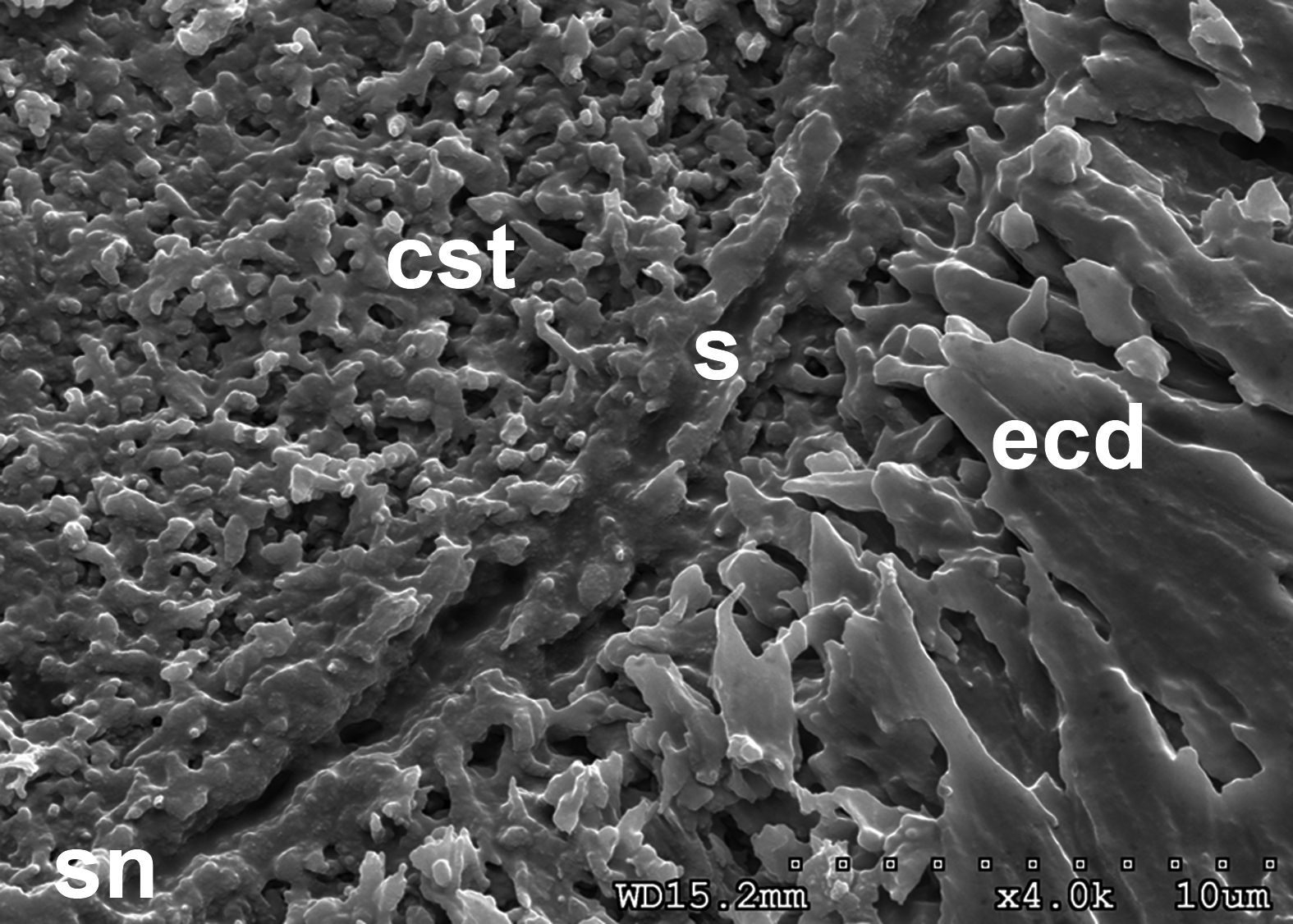

Supplement: S3 Fig — NRM–PZ Mo 167764. The traces of thin organic structure-less septum squeezed between prismatic episeptal cameral deposits (right side) and granular hyposeptal cameral soft tissue remains (left side) at septal neck. Cst, cameral soft tissue remains; ecd, episeptal cameral deposits; s, septum; sn, septal neck. (TIF) [file pone.0172169.s004.tif]

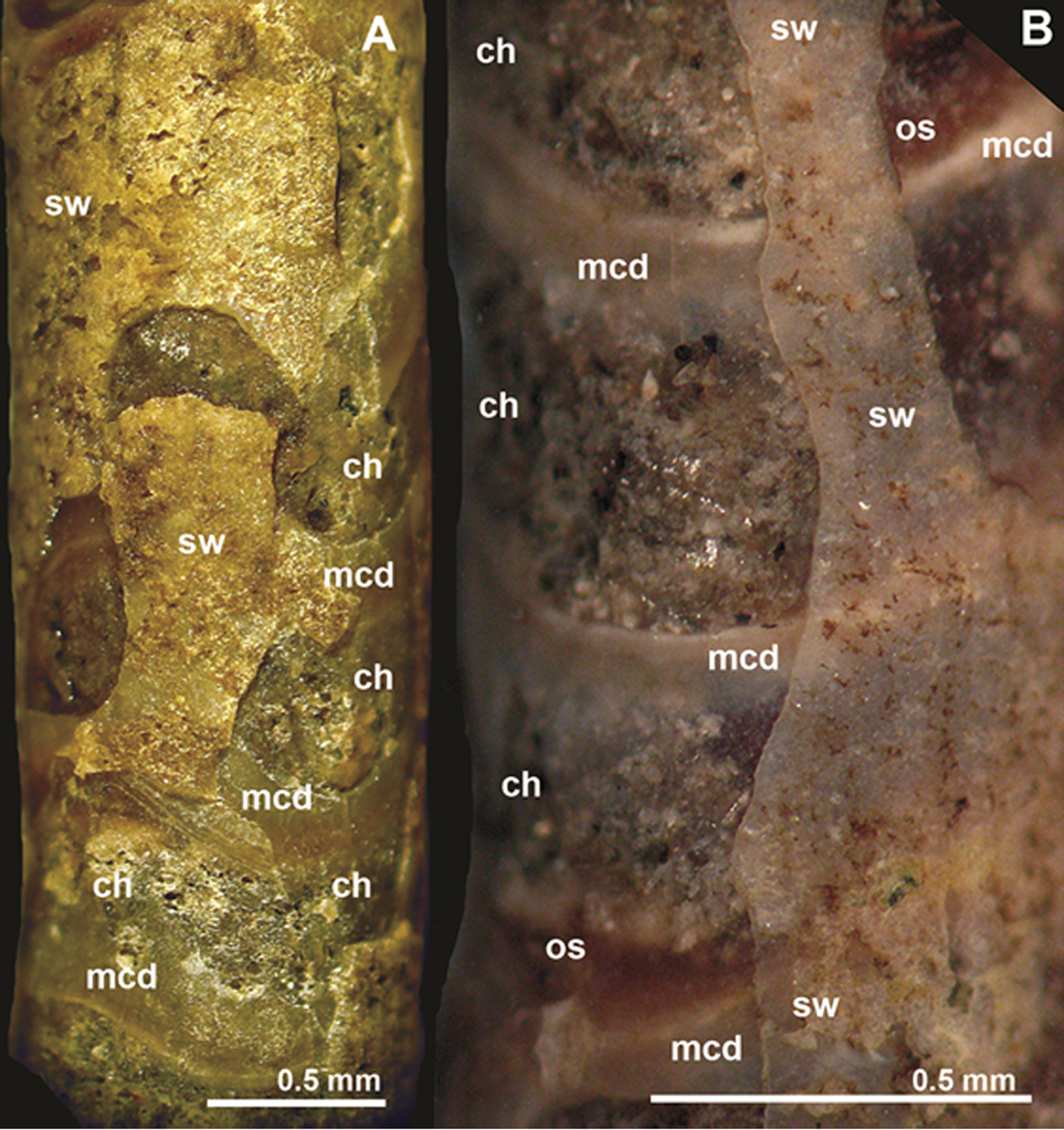

Supplement: S4 Fig — NRM–PZ Mo 167765. A, Ventrolateral view on the partially exposed chambers of the phragmocone with extensive mural cameral deposits. B, Enlargement of Fig 1E to show remains of thin brown organic septa. C, chamber; mcd, mural cameral deposits; os, organic septum; sw. shell wall. (TIF) [file pone.0172169.s005.tif]

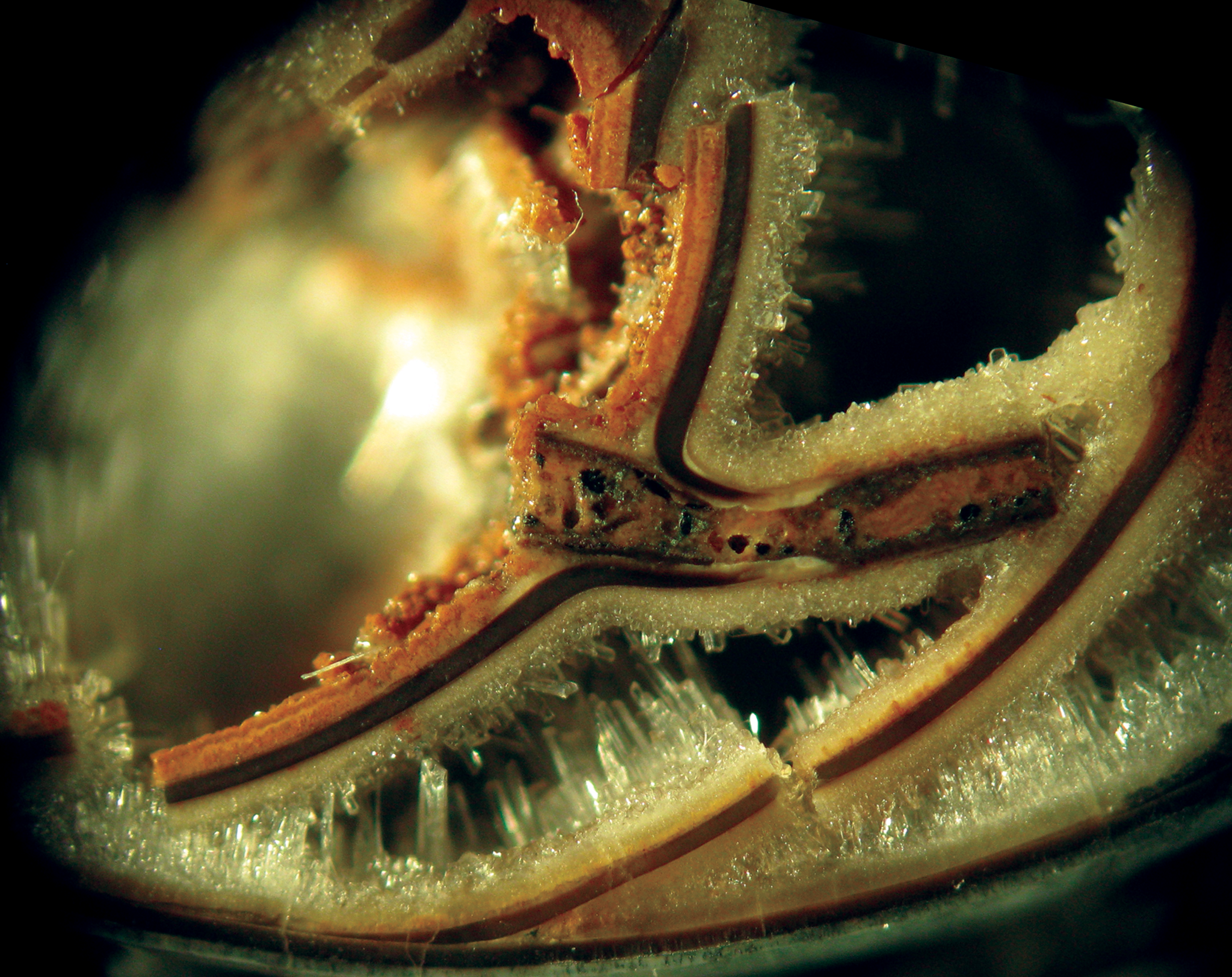

Supplement: S5 Fig — NRM–PZ Mo 167766. Median shell section showing sub-central narrow siphuncle. (TIF) [file pone.0172169.s006.tif]

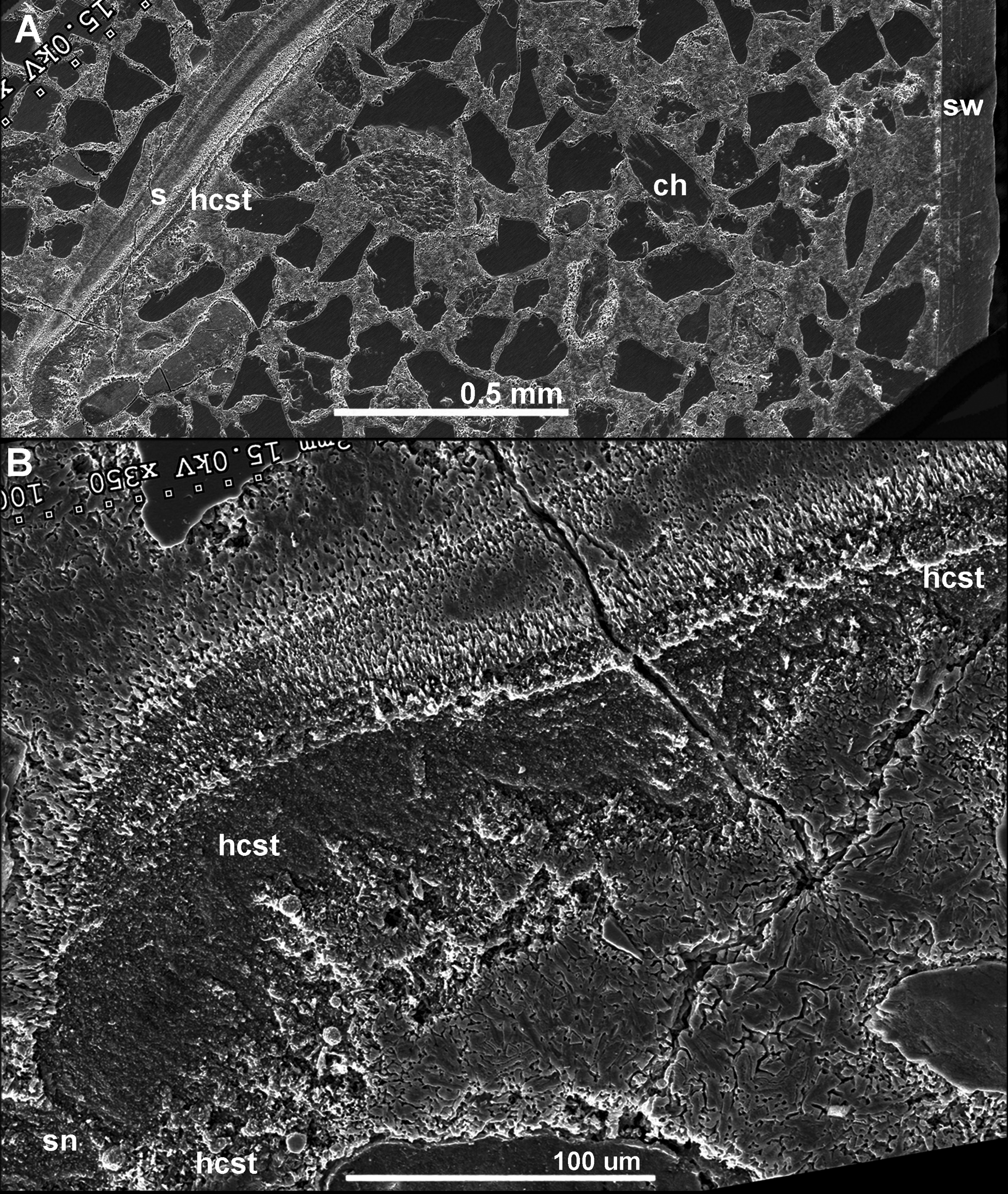

Supplement: S6 Fig — NRM–PZ Mo 167764–2. A, hyposeptal cameral soft tissues lining the adapical septal surface. B, enlargement of A showing thin suborthochoanitic septal neck and microglobular ultrastructure of hyposeptal cameral soft tissue. Ch, chamber; hcst, hyposeptal cameral soft tissue; s, septum; sn, cyrtochoanitic septal neck. (TIF) [file pone.0172169.s007.tif]

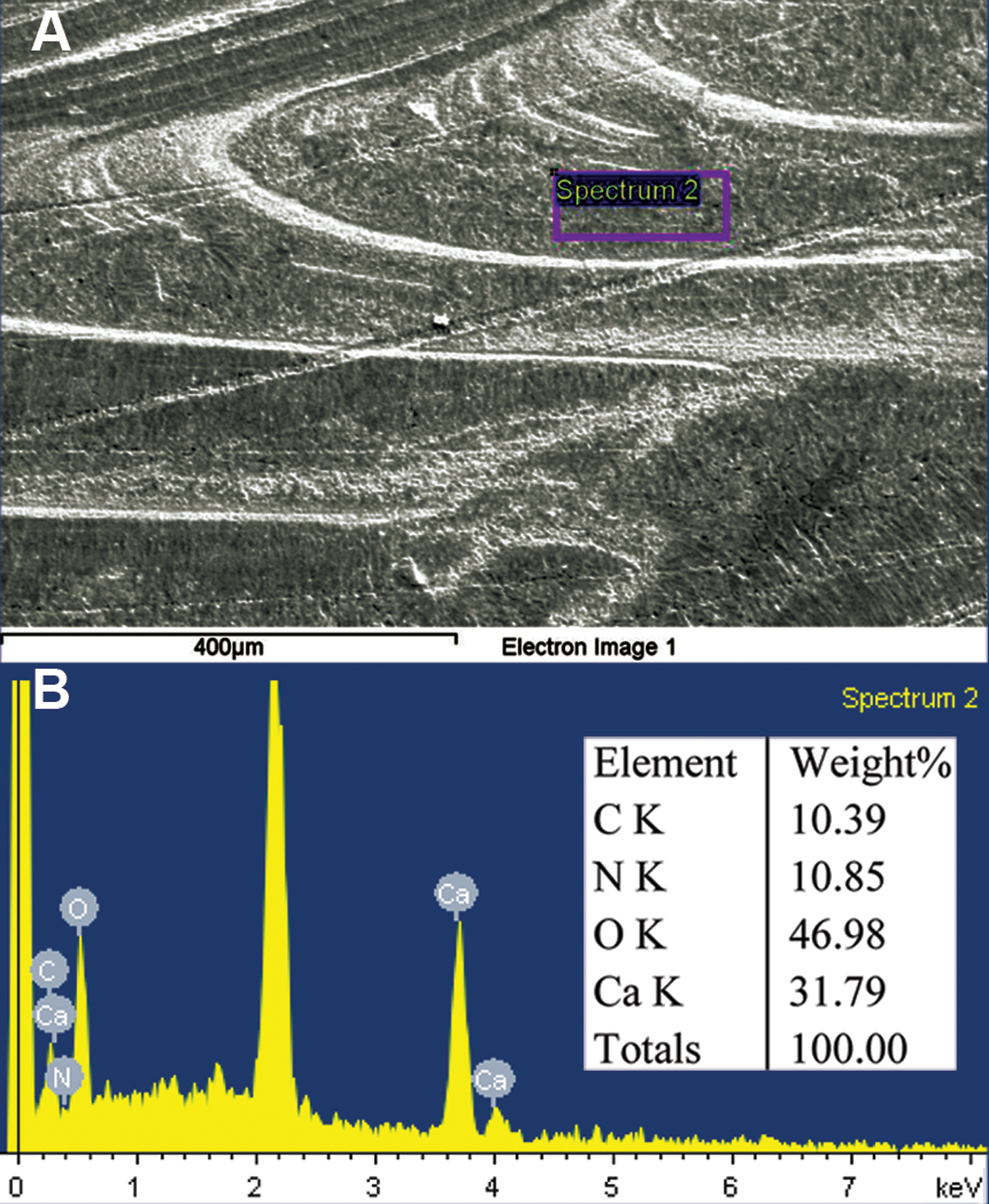

Supplement: S7 Fig — Note a peak of nitrogen indicative of non-fossilized organic material [57]. (TIF) [file pone.0172169.s008.tif]

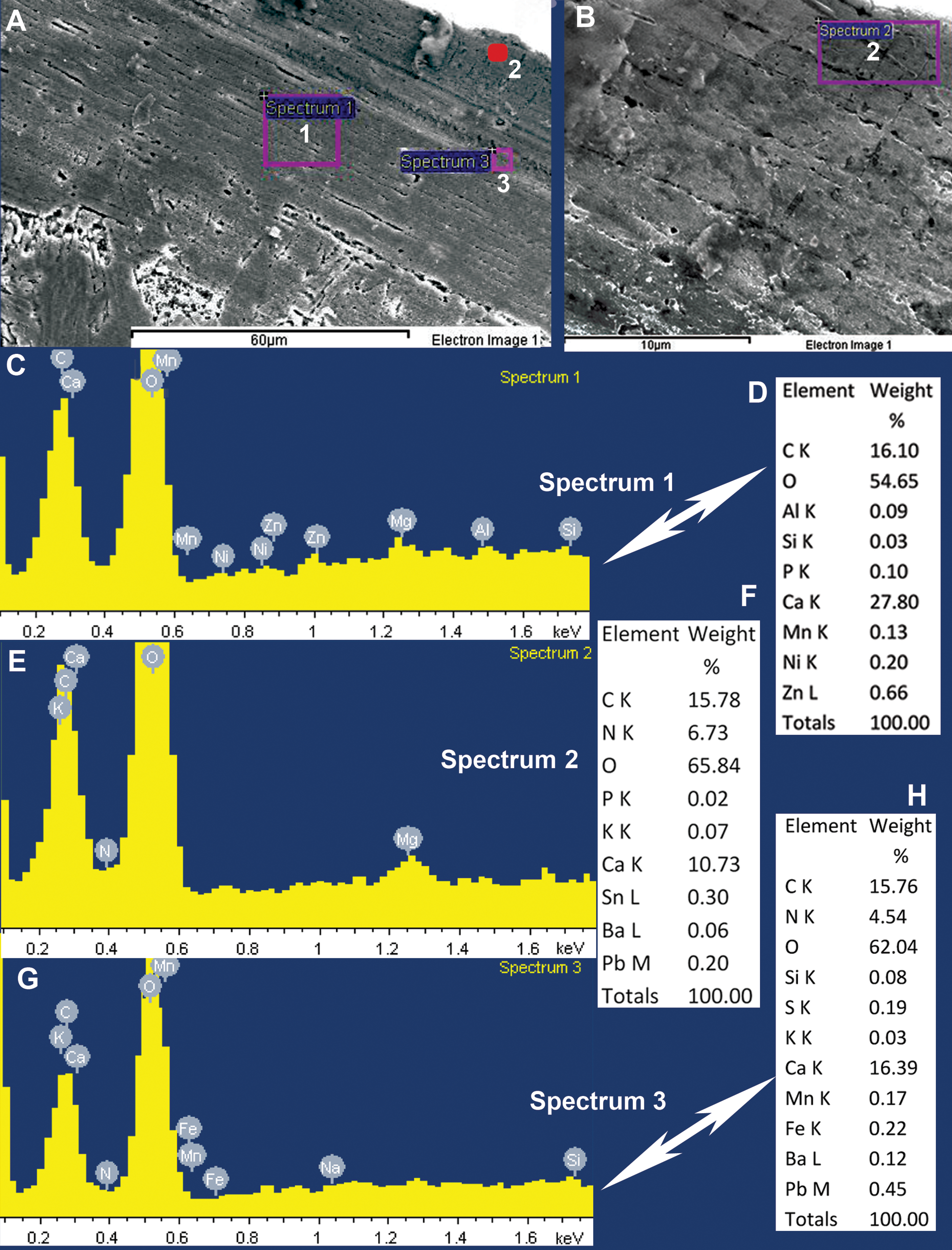

Supplement: S8 Fig — NRM–PZ Mo 167764. Energy-dispersive spectrometry data on chemical composition of shell wall (in percent of total weight). (TIF) [file pone.0172169.s009.tif]

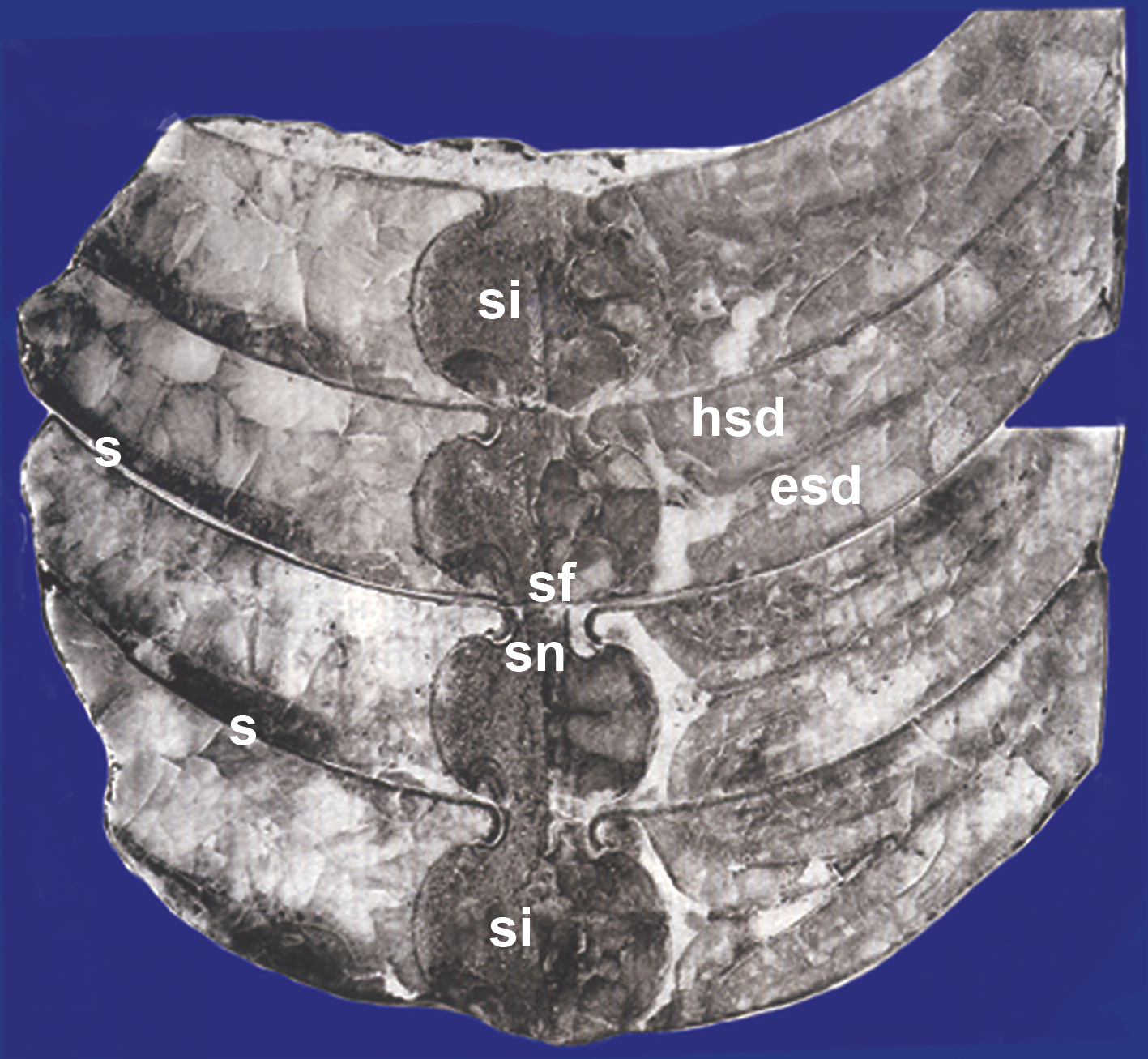

Supplement: S9 Fig — Median shell section ([43]; modified). esd, episeptal cameral deposits; hsd, hyposeptal cameral deposits; s, septum; sf, siphuncular foramen; si, siphuncle; sn, septal neck. (TIF) [file pone.0172169.s010.tif]

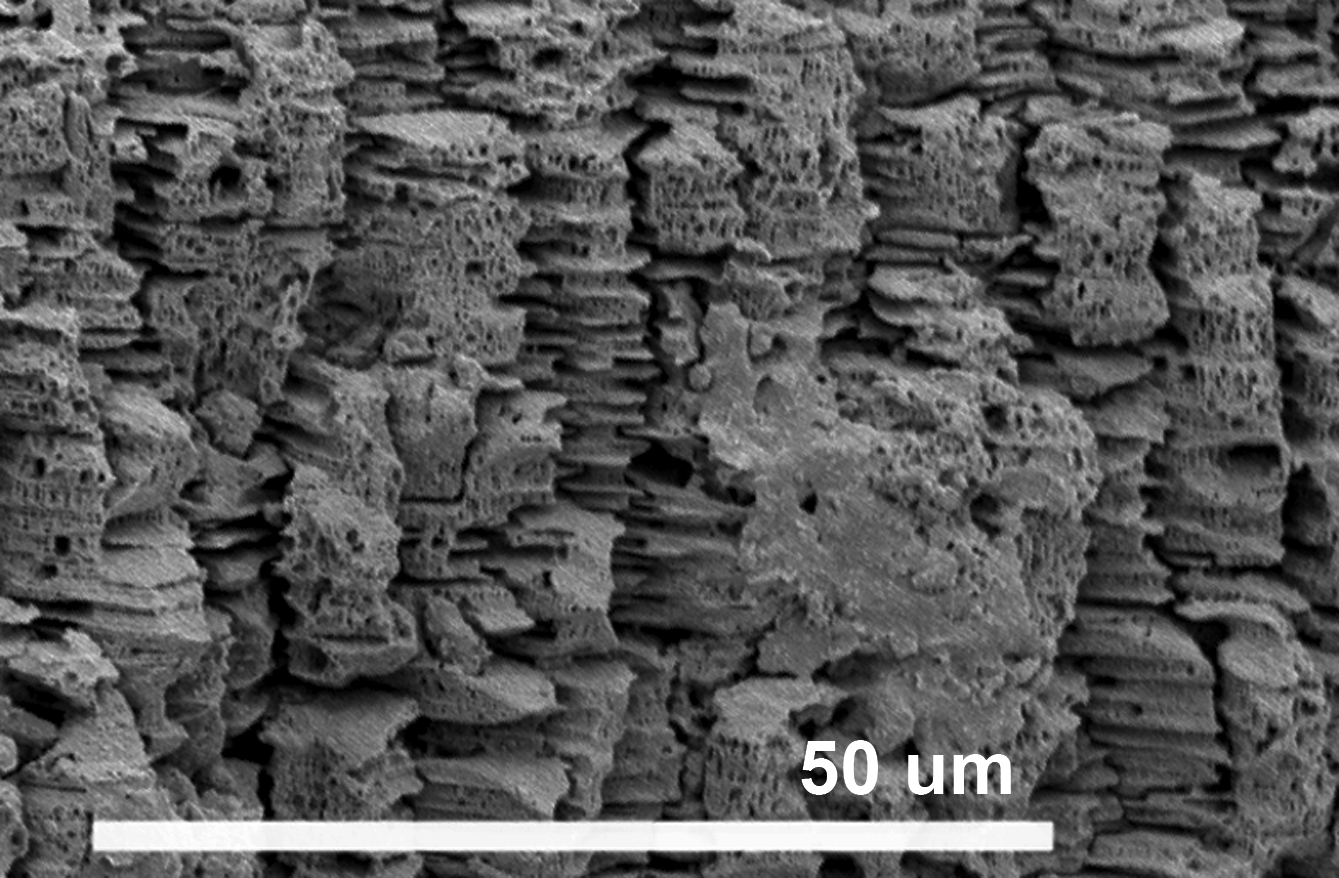

Supplement: S10 Fig — NRM–PZ Mo 167766. Columnar nacre of shell wall, median shell section. (TIF) [file pone.0172169.s011.tif]

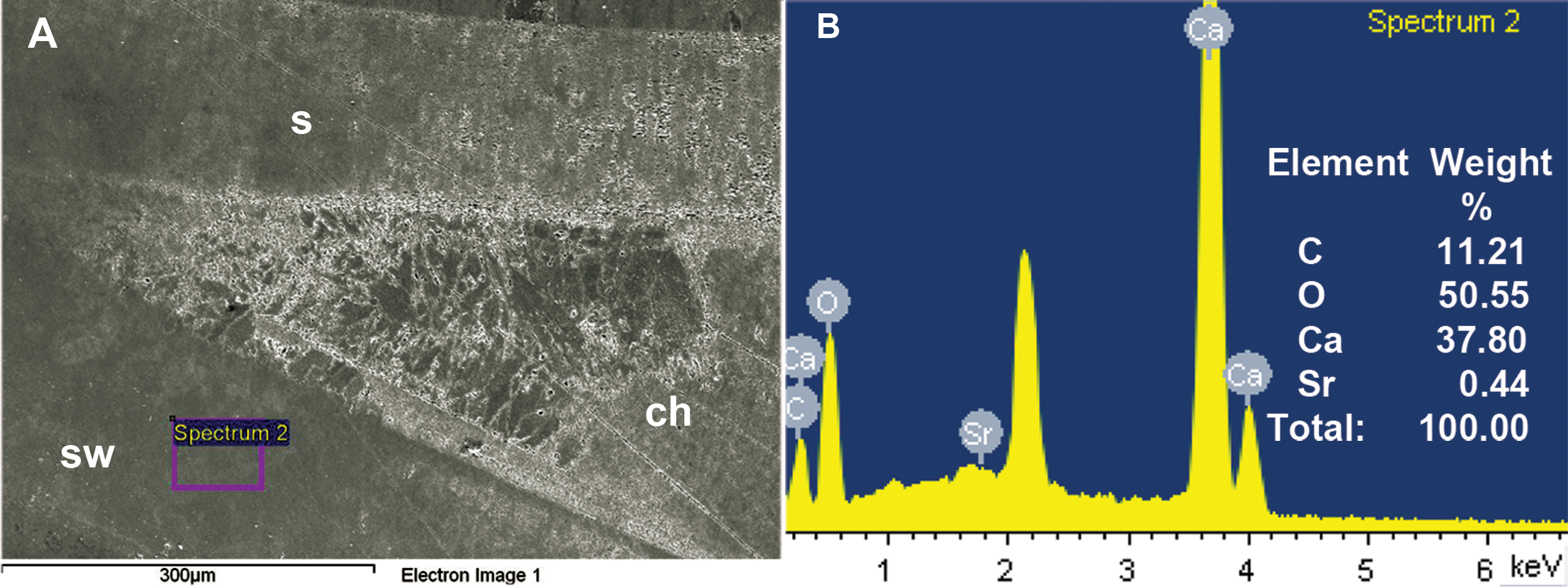

Supplement: S11 Fig — NRM–PZ Mo 167766. Energy-dispersive spectrometer data on shell wall chemical composition. A, median shell section showing position of the spectrum taken. Ch, chamber of the phragmocone; s, septum; sw, shell wall. B, An energy-dispersive spectrometer graph showing spectrum 2 in A. (TIF) [file pone.0172169.s012.tif]
